# Predictors of intensive care unit admission and mortality in patients with ischemic stroke: investigating the effects of a pulmonary rehabilitation program

**DOI:** 10.1186/s12883-017-0912-4

**Published:** 2017-07-11

**Authors:** Belma Doğan Güngen, Abdulkadir Tunç, Yeşim Güzey Aras, Aslı Aksoy Gündoğdu, Adil Can Güngen, Serdar Bal

**Affiliations:** Bezmialem Vakif Universitesi Tip Fakultesi Hastanesi, Istanbul, Turkey

**Keywords:** Stroke, Mortality, Intensive care unit, Pulmonary rehabilitation

## Abstract

**Background:**

The aim of this study was to investigate the predictors of intensive care unit (ICU) admission and mortality among stroke patients and the effects of a pulmonary rehabilitation program on stroke patients.

**Methods:**

This prospective study enrolled 181 acute ischemic stroke patients aged between 40 and 90 years. Demographical characteristics, laboratory tests, diffusion-weighed magnetic resonance imaging (DWI-MRI) time, nutritional status, vascular risk factors, National Institute of Health Stroke Scale (NIHSS) scores and modified Rankin scale (MRS) scores were recorded for all patients. One-hundred patients participated in the pulmonary rehabilitation program, 81 of whom served as a control group.

**Results:**

Statistically, one- and three-month mortality was associated with NIHSS and MRS scores at admission and three months (*p*<0.001; *r*=0.440, *r*=0.432, *r*=0.339 and *r*=0.410, respectively). One and three months mortality- ICU admission had a statistically significant relationship with parenteral nutrition (*p*<0.001; *r*=0.346, *r*=0.300, respectively; *r*=0.294 and *r*=0.294, respectively). Similarly, there was also a statistically significant relationship between pneumonia onset and one- and three-month mortality- ICU admission (*p*<0.05; *r*=0.217, *r*=0.127, *r*=0.185 and *r*=0.185, respectively). A regression analysis showed that parenteral nutrition (odds ratio [OR] =13.434, 95% confidence interval [CI] =1.148–157.265, *p*=0.038) was a significant predictor of ICU admission. The relationship between pulmonary physiotherapy (PPT) and ICU admission- pneumonia onset at the end of three months was statistically significant (*p*=0.04 and *p*=0.043, respectively).

**Conclusion:**

This study showed that PPT improved the prognosis of ischemic stroke patients. We believe that a pulmonary rehabilitation program, in addition to general stroke rehabilitation programs, can play a critical role in improving survival and functional outcomes.

**Trial registration:**

NCT03195907. Trial registration date: 21.06.2017 ‘Retrospectively registered’.

## Background

Severe stroke remains an important cause of mortality and morbidity, despite advances in disease management, acute treatment and secondary measures [1]. Predicting early mortality and disability after a stroke depends on many factors, such as age, the type of stroke, lesional location, level of consciousness, severity of neurological impairment, medical risk factors (hypertension and diabetes), premorbid conditions, fever and history of stroke [2]. Stroke patients may experience a reduction of up to 50% in respiratory function when compared to age- and gender-matched norms [3]. The reduction in respiratory function can lead to decreased endurance, dyspnoea and increased sedentary behaviour, as well as an elevated risk of stroke. The reduction in respiratory function may also cause aspiration, leading to pneumonia [4]. Previous research showed that pneumonia was an independent risk factor for mortality and a poor prognosis in stroke patients [1]. Research also showed that a reduction in respiratory muscle and abdominal muscle strength contributed to pulmonary and respiratory dysfunction following a stroke. Low respiratory muscle function decreases the efficacy of rehabilitation because it leads to exercise intolerance in stroke patients [5]. Thus, special exercise programs are needed to improve the pulmonary function and respiratory muscle strength of stroke patients.

The aim of pulmonary rehabilitation program is to enhance respiratory muscle resistance during breathing, thereby improving respiratory function. Previous studies demonstrated that pulmonary rehabilitation programs improved respiratory functions in cardiac disease and chronic obstructive pulmonary disease patients [6, 7]. There has been little research on the use of pulmonary rehabilitation programs in stroke patients. McCool and Tzelepis advised that respiratory muscle strength and endurance increased when initial stroke patients performed exercise at sufficient intensity to increase muscle strength, so that eventual improvement in respiratory functions could be expected [8]. In addition, pulmonary rehabilitation programs were considered to be capable of inducing positive effects on stroke patients’ respiratory muscles through diaphragm breathing exercise and lip puckering breathing exercise in another study [9]. Another study showed that pulmonary physiotherapy (PPT) improved the quality of life of stroke patients [4].

The aims of the present study were to investigate the predictors of intensive care unit (ICU) admission and mortality among stroke patients and the effects of PPT on these stroke patients.

## Methods

This prospective study enrolled 181 acute ischemic stroke patients aged between 40 and 90 years who were admitted to the neurology clinic stroke unit or the neurological ICU of the Sakarya University Training and Research Hospital between February 2015 and January 2016. The inclusion criteria were acute ischemic stroke confirmed by computed tomography (CT) or a diffusion-weighed magnetic resonance imaging (DWI-MRI) scan, aged > 40 years, ability to understand and follow simple verbal instructions, modified Rankin scale (MRS) score > 2, National Institute of Health Stroke Scale (NIHSS) score > 0, no unrestricted movement of the lips, no receptive aphasia and no history of thoracic or abdominal surgery. The exclusion criteria were as follows: blood pressure >180/100 mm Hg more than twice in 24 h; significant pulmonary disease, angina, myocardial infarction or acute heart failure within three months; neurological conditions other than stroke; presence of a severe visual disability and visual field defects; receiving medications that would influence the metabolic or cardiorespiratory responses to exercise; inability to perform the tests.

This study was approved by Sakarya University Human Ethics Committee (Reference number: 16214662/050.01.04/154). Prior to participation in the study, each individual completed a detailed written informed consent form.

### Data collection

Demographical characteristics, laboratory tests, DWI-MRI time and nutritional status were recorded for all patients. Vascular risk factors, including hypertension, diabetes, smoking status and alcohol consumption, were evaluated. NIHSS and MRS scores were used at baseline and three months to assess stroke severity and functional disability [10, 11]. Infarct sizes and locations were recorded based on cranial MRI findings. Lesional areas were categorised according to the areas of the vessels: medial cerebral artery (MCA), anterior cerebral artery, posterior cerebral artery, basilar artery and cerebellar arteries. The MCA infarcts were further classified according to the dimensions of the lesions (MCA1: lacunar infarcts; MCA2: between lacunar infarcts and total MCA infarcts; and MCA3: total MCA infarcts).

The subtypes of ischemic stroke were classified according to the original Trial of Org 10172 Acute Stroke Treatment (TOAST) criteria [12]. The five major categories of the TOAST classification are: large artery atherosclerosis, including large artery thrombosis and artery-to-artery embolisms, cardioembolisms, small artery occlusions, stroke of other determined cause and stroke of undetermined cause. The definitions of the subtypes were made according to risk factor profiles, clinical features and results of diagnostic tests. CT, MRI, vascular imaging (carotid-vertebral artery Doppler ultrasound), electrocardiograms and echocardiography were performed, and pro-thrombotic syndromes were assessed.

### Stroke management and three-month follow up

Stroke patients were transferred from the emergency unit to the stroke unit within 24 h. Stroke management was based on the guidelines of the American Heart Association/American Stroke Association [13]. Various measures were implemented. These included providing supportive treatment to ensure a patent airway, maintaining oxygen saturation above 92%, maintaining haemodynamic stability, providing fever and glycaemic control, providing hydration and nutrition and preventing pressure sores and deep venous thrombosis [2]. Antiplatelet agents (aspirin and clopidogrel) were used for the treatment of ischemic stroke [14]. Patients who required mechanical ventilation were admitted to the neurological ICU. The patients were followed up at the hospital or after discharge for a three-month period. Mortality and admission to the ICU were recorded at the end of the first and third months.

### Pulmonary rehabilitation program

Patients were assigned randomly to either the PPT or control group by having each of the subjects take out 1 card from a box containing 2 types of cards representing both study groups. One-hundred patients participated in the pulmonary rehabilitation program (group 1), and 81 patients served as a control group (group 2). Both groups participated in a daily conventional rehabilitation program, which included joint mobility, eccentric contraction, muscle strengthening and walking exercises. Only the patients in group 1 participated in the PPT program. The program was conducted by physical therapists at our hospital for 30 min, three days/week. As part of the PPT, a physiotherapist monitored group 1 for 12 weeks. The same physiotherapist supervised all the exercises.

During the exercise program, all patients were clinically stable and all were receiving optimal medical therapy. Rehabilitation started with inspiratory diaphragm breathing exercises. The physiotherapist placed his hands on the superior rectus abdominis immediately below the anterior costal cartilage and induced inspiratory diaphragm breathing by instructing the patient to slowly and deeply inhale the air through the nose. Then the patient was instructed to perform expiratory pursed-lip breathing exercise by continuously exhale the air. During pursed-lip breathing exercise, the patient was instructed in sequence, to breathe in gently through the nose, purse his/ her lips as though whistling and then breathe out through the long pursed lips by not exerting power until she/ he is short of breath. The expiration time was set to be at least twice times longer than inspiration time [9, 15]. The patients took a rest when they complained about fatigue or dizziness during breathing exercise and conducted breathing exercise again.

The exercise intensity was based on the maximal heart rate and maximal effort of the patients. The exercise intensity was increased gradually over the course of the 12-week program. Each patient’s performance during the exercise sessions was recorded and reported regularly to the patient’s physician. NIHSS scores, MRS scores, pneumonia onset, admission to the ICU and mortality in groups 1 and 2 were recorded at the end of the first and third month.

### Statistical analysis

Statistical analyses were carried out using SPSS/PC software (version 21.0). Descriptive statistics were presented as mean values, with standard deviations for numerical variables. Independent *t* tests were conducted to examine the difference between categorical variables in the two groups, and a one-way analysis of variance was used for examining the difference between the categorical variables in the two groups. A chi square test was used to determine the correlation between two categorical variables, and Pearson’s correlation coefficients were used to examine the correlation between two numerical variables. The limit for statistical significance was accepted as *p*<0.05. To investigate the effect of the independent variables on the dependent variable (ICU admission), multiple linear regression analyses were used.

## Results

As shown in Table 1, there were no significant differences in the baseline values of the two groups as regards their general characteristics, vascular risk factors, feeding type, NIHSS and MRS scores, and MRI findings and classification based on TOAST criteria (*p*>0.05). Group 1 had a mean age of 68.94±12.5 years, and 39 (21.5%) of the patients were males. In group 2, the mean age was 70.95±12.8 years, and 40 (22.7%) of the patients were males. The NIHSS score on admission was 6.53±5.038 in group 1, and it was 6.72±5.028 in group 2 (*p*=0.805).

**Table 1:** Demographic and clinical characteristics of the patients

		Stroke patients		*P* value*
		Pulmonary rehabilitation (+) Group 1 (n=100)	Pulmonary rehabilitation (-) Group 2 (n=81)	
Age (year)		68.94±12.505	70.95±12.875	0.290
Sex				
Male		39(21.5%) /	40(22.7%) /	0.161
Female		61(33.7%)	41(22.1%)	
Alcohol consumption		4(2.2%)	1(0.6%)	0.259
Smoking		27(14.9%)	17(9.4%)	0.201
Hypertension		69(38.1%)	59(32.6%)	0.421
Diabetes		39(21.5%)	26(14.4%)	0.336
WBC (×10^9^/L)		7.54±2.199	8.16±3.312	0.068
Neutrophil count (×10^9^)		4.54±1.957	4.24±1.714	0.292
Platelets		233.40±80.346	234.20±96.881	0.952
CRP (mg/L)		17.65 ±26.984	12.93±20.624	0.197
ESR(mm/hr)		31.30±23.6	28.51±20..81	0.410
Nasogastric feeding		13(7.2%)	16(8.8%)	0.218
Parenteral Nutrision		13(7.2%)	16(8.8%)	0.218
DWI time (hour)		9.96±5.151	10.34±4.069	0.595
NIHSS score at admission		6.53±5.038	6.72±5.028	0.805
Infarction size (cm)	0.5-1.5	31(17.1%)	23(12.7%)	0.914
	1.5-5	55(30.4%)	47(20.6%)	
	≥ 5	14(7.7%)	11(6.1%)	
MRS score	0	5(2.8%)	1(0.6%)	
	1	33(18.2%)	31(17.1%)	
	2	28(15.1%)	19(10.5%)	
	3	17(9.4%)	16(8.8%)	
	4	13(7.2%)	7(3.9%)	
	5	4(2.2%)	5(2.8%)	
	6	0	2(1.1%)	
Cranial magnetic resonance findings	MCA 1	24(13.3%)	18(9.9%)	0.511
	MCA 2	34(18.8%)	26(14.4%)	
	MCA 3	5(2.8%)	4(2.2%)	
	ACA	2(1.1%)	1(0.6%)	
	PCA	22(12.2%)	12(6.6%)	
	Basilary artery	8(4.4%)	10(5.5%)	
	Cerebellar artery	5(2.8%)	10(5.5%)	
TOAST criteria	large-artery atherosclerosis	30(16.6%)	25(13.8%)	0.751
	cardioembolism	31(17.1%)	23(12.7%)	
	small artery occlusion	24(13.3%)	24(13.3%)	
	other determined cause	4(2.2%)	4(2.2%)	
	undetermined cause	11(6.1%)	5(2.8%)	

Data are mean (SD) and frequency (%), unless stated otherwise. *WBC* white blood cells, *CRP* C-reactive protein (normal <0,9), *ESR* erythrocyte sedimentation rate (normal <20), *NIHHS* National Institute of Health Stroke Scale, *TOAST* Trial of Org 10172 in Acute Stroke Treatment, *DWI* diffusion-weighted MR imaging, *MCA* medial cerebral artery, *ACA* anterior cerebral artery, *PCA* posterior cerebral artery, *MRS* modified Rankin scale, *SD* standard deviation

* *p* < 0.05 ; statistical significance, with Student’s t test

### ICU admission and mortality

Analysis of the effects of relevant determinants on one- and three-month mortality and ICU admission revealed no statistically significant effects of age, gender, hypertension, diabetes, nasogastric feeding, lesional areas on MRI and lesional subtypes according to TOAST criteria (*p*>0.05).

Statistically, one- and three-month mortality was associated with NIHSS and MRS scores at admission and three months (*p*<0.001; *r*=0.440, *r*=0.432, *r*=0.339 and *r*=0.410, respectively). No significant relationship was found between these parameters and ICU admission (*p*>0.05). One and three months mortality- ICU admission had a statistically significant relationship with parenteral nutrition (*p*<0.001; *r*=0.346, *r*=0.300, respectively; *r*=0.294 and *r*=0.294, respectively). Similarly, there was a statistically significant relationship between pneumonia onset and mortality- ICU admission at the end of one and three months (*p*<0.05; *r*=0.217, *r*=0.127, *r*=0.185 and *r*=0.185, respectively) (Table 2).

**Table 2:** The effects of relevant determinants on 1 and 3-months mortality and intensive care unit admission

	Stroke patients		1^st^ month mortality	3^th^ month mortality
	ICU admission at 1^st^ month	ICU admission at 3^th^ month		
Age	r:0.064	r:0.121	r:0.11	r:0.111
	p:0.389	p:0.104	p:0.135	p:0.135
Sex (Male)	r:0.028	r:0.066	r:0.056	r:0.056
	p:0.713	p:0.378	p:0.457	p:0.457
Hypertension	r:0.073	r:0.049	r:0.030	r:0.030
	p:0.329	p:0.511	p:0.690	p0.690
Diabetes	r:0.049	r:0.039	r:0.014	r:0.014
	p:0.513	p:0.331	p:0.848	p:0.848
Cranial magnetic resonance findings	r:0.138	r: 0.039	r:0.140	r:0.140
	p:0.065	p: 0.601	p:0.068	p:0.068
NIHSS score at admission	r:0.210	r:0.323	r:0.440	r:0.440
	p:0.005	p:0.001	p: <0.001	p: <0.001
NIHSS score at 3 months	r:0.276	r:0.355	r:0.432	r:0.432
	p:0.001	p:0.001	p: <0.001	p: <0.001
MRS score at admission	r:0.197	r:0.136	r:0.339	r:0.339
	p:0.008	p:0.069	p: <0.001	p: <0.001
MRS score at 3 months	r:0.195	r:0.169	r:0.410	r:0.410
	p:0.008	p:0.026	p: <0.001	p: <0.001
Nasogastric feeding	r:0.228	r:0.061	r:0.323	r:0.323
	p:0.002	p:0.415	p:0.001	p:0.001
Parenteral nutrision	r:0.346	r:0.300	r:0.294	r:0.294
	p:0.001	p:0.001	p:0.001	p:0.001
Pneumonia	r:0.217	r:0.127	r:0.185	r:0.185
	p:0.003	p:0.089	p:0.013	p:0.013
TOAST criteria	r:0.079	r:0.047	r:0.047	r:0.047
	p:0.292	p:0.530	p:0.530	p:0.530

ICU, intensive care unit; NIHHS, National Institute of Health Stroke Scale; MRS, modified Rankin scale; TOAST, Trial of Org 10172 in Acute Stroke Treatment

*p* < 0.05; r, correlation coefficient

The multiple logistic regression using the potential variables (NIHSS scores, MRS scores, pneumonia, nasogastric feeding and parenteral nutrition) from the bivariate analysis was adjusted to detect predictors of ICU admission. The regression analysis showed that parenteral nutrition (odds ratio [OR]: 13.434, 95% confidence interval [CI]: 1.148–157.265, *p*=0.038) was a significant predictor of ICU admission (Table 3).

**Table 3:** Multivariate logistic regression model for intensive care unit admission

	β	S.E. of β	*p* values*	OR (95% C.I.)
NIHSS score at admission	0.165	0.182	0.365	0.848 (0.593-1.212)
NIHSS score at 3 months	0.297	0.187	0.112	1.345 (0.933-1.943)
MRS score at admission	0.341	0.543	0.530	0.711 (0.245-2.060)
MRS score at 3 months	0.133	0.397	0.737	0.875 (0.402-1.905)
Pneumonia	0.648	1.085	0.550	1.912 (0.228-16.033)
Nasogastric feeding	0.211	1.309	0.872	0.810 (0.062-10.539)
Parenteral nutrision	2.598	1.255	0.038	13.434 (1.148-157.265)

*Abbreviations*: *NIHHS* National Institute of Health Stroke Scale, *MRS* Modified Rankin scale, *OR* Odds ratio, *CI* Confidence interval, *SE* Standart error

* *p* < 0.05

### Effects of the pulmonary rehabilitation program

The three-month NIHSS scores were statistically significantly lower compared with the baseline scores in both groups (*p*<0.001). The three-month MRS scores were statistically significant in group 1 compared with the baseline scores (*p*<0.001). When the effect of the PPT program on one- and three- month mortality was examined, no significant difference was found in either group at the end of 1 month (0%). However, at the end of three months, the mortality in group 1 (*n* = 2, 2%) was slightly lower than that in group 2 (*n* = 3, 3.7%), as shown in Table 4.

**Table 4:** Values of NIHSS- MRS scores at admission and 3 months and mortality- ICU admission at 1 and 3 months in both groups

PPT group				p*
NIHSS score at admission	6.53 ±5.038	NIHSS score at 3 months	5.13 ±5.198	<0.001
MRS score at admission	3.2	MRS score at 3 months	2.5	<0.001
ICU admission at 1^st^ month	3(3.0%)	ICU admission at 3 months	1(1.0%)	
1 month mortality	0(0%)	3 months mortality	2(2%)	
Control group				
NIHSS score at admission	6.72 ±5.028	NIHSS score at 3 months	5.86 ±5.239	0.002
MRS score at admission	2.3	MRS score at 3 months	2.04	0.507
ICU admission at 1^st^ month	4(4.9%)	ICU admission at 3 months	5(6.2%)	
1 month mortality	0(0%)	3 months mortality	3(3.7%)	

*Abbreviations*: *PPT* Pulmonary physiotherapy, *ICU* Intensive care unit, *NIHHS* National Institute of Health Stroke Scale, *MRS* Modified Rankin scale

* *p* < 0.05

The relationship between PPT and ICU admission and pneumonia onset at the end of three months was statistically significant (*p*=0.04 and *p*=0.043, respectively). In group 1, five (2.8%) patients had pneumonia, and one (1.0%) patient was admitted to the ICU. In group 2, 11 (6.1%) patients had pneumonia, and five (6.2%) patients were admitted to the ICU (Table 5).

**Table 5:** Effects of pulmonary rehabilitation on mortality, pneumonia onset, ICU admission, NIHSS and MRS scores at 3 months

	PPT group	Control group	p*
NIHSS score at 3 months	5.13 ±5.198	5.86 ±5.239	0.348
MRS score at 3 months	2.5	2.04	0.211
ICU admission at 3 months	1(1.0%)	5(6.2%)	0.04
3 months mortality	2(2%)	3(3.7%)	0.507
3 months pneumonia onset	5(2.8%)	11(6.1%)	0.043

*PPT* Pulmonary physiotherapy, *ICU* Intensive care unit, *NIHHS* National Institute of Health Stroke Scale; *MRS* Modified Rankin scale

* *p* < 0.05

## Discussion

This study investigated the predictors of ICU admission and mortality and the effects of PPT on stroke patients. The results showed that the pulmonary rehabilitation program reduced ICU admission and pneumonia onset rates at the end of three months. They also revealed a statistically significant relationship between one- and three-month mortality and NIHSS and MRS scores at admission and three months and a statistically significant relationship between one- and three-month mortality and parenteral nutrition and pneumonia. Similarly, the relationship between parenteral nutrition, pneumonia onset and ICU admission was statistically significant. The regression analysis showed that parenteral nutrition was a significant predictor of ICU admission.

Based on a recent comparison of stroke results in different European regions, neurological impairment, a poor prognosis or mortality was recorded in 40% of patients at the end of the three months following the first episode of stroke [16]. In that study, age, stroke severity, comorbid factors and living pre-stroke dependency were determinants of post-stroke mortality and poor outcomes. Early post-stroke complications, such as pneumonia and increased intracranial pressure, were also reported to be independent risk factors for mortality and poor functional outcomes [17].

In stroke patients, the leading indications for ICU admission were reported to be deterioration of neurological status, thrombolytic treatment requirements, brain stem infractions and large hemispheric infarcts [18]. Other indications for ICU admission were a fluctuating clinical course, haemodynamic instability, multiple or septic emboli, significant cardiac dysrhythmia, respiratory and metabolic instability and the need for mechanical ventilation [18].

A previous study demonstrated that NIHSS and MRS scores were associated with the three-month prognosis in stroke patients [19]. In a study in Nepal, the NIHSS score at admission predicted seven-day mortality in ICUs, but the study did not evaluate long-term predictors of outcomes in acute stroke patients [20]. In the present study, the relationship between NIHSS and MRS scores at admission and three months and mortality rates at the end of one and three months were statistically significant, but there was no relationship between these scores and ICU admission. The relationship between NIHSS and MRS scores and mortality is concordant with that reported in previous studies [19, 20]. However, unlike the literature, these scores had no prognostic value as regards ICU admission. We attributed this finding to the limited number of patients and the effects of comorbid factors.

In the present study, the relationship between parenteral nutrition and mortality and ICU admission was statistically significant, as was the relationship between pneumonia onset and mortality and ICU admission. Based on the multivariable analysis, parenteral nutrition increased the rates of ICU admission 13-fold. A stroke causes neurogenic oropharyngeal dysphagia, which limits food intake, considerably increasing the risk of malnutrition and tracheal aspiration, and triples the incidence of aspiration pneumonia. As reported in the literature, neurogenic oropharyngeal dysphagia occurred in about 25–90% of stroke patients, and swallowing disorders, frequently accompanied by tracheal aspiration, occurred in 45% of stroke patients [21]. Aspiration pneumonia was reported to develop in 50% of patients who suffered from tracheal aspiration [21]. The Berlin Stroke Register analysis demonstrated that pneumonia onset increased in-hospital death five-fold [22]. In the current study, the patients were received enteral feeding whenever possible. The patients with suspected aspiration or severe impaired consciousness were received parenteral nutrition, as patients receiving parenteral nutrition are expected to have a poor prognosis. In accordance with the previous studies [17, 22], pneumonia onset was also associated with a poor prognosis in the present study.

The cardiopulmonary functions of stroke patients are diminished due to damage of the hemi-thorax and deterioration of respiratory muscles [23]. As a result of the paralysis of the diaphragm and respiratory muscles following a stroke, the thorax cannot expand sufficiently, which can lead to shortening of the thoracic cells and muscle fibrosis. The expansion capacity of the thorax may consequently be reduced during breathing [24]. In addition, cardiorespiratory control and oxygen-transferring systems are impaired in stroke patients due to limited muscular movement. The latter has been associated with a lack of oxygen and increased metabolic demands [23]. The deterioration in cardiopulmonary function is a leading cause of morbidity in stroke patients [24].

Previous research reported that aerobic exercise decreased cardiovascular risk factors, in addition to improving cardiopulmonary functions and functionality in activities of daily life and enhancing motor-sensory functions, leading to improved walking abilities in stroke patients [25]. General stroke rehabilitation programs that focus only on physical and functional recovery may not adequately enhance cardiopulmonary function in stroke patients [23].

The present study demonstrated that PPT reduced ICU admission and pneumonia onset rates at the end of three months. The positive prognostic effect of the PPT program is in accordance with that found in previous studies [23–25]. The results suggest that a pulmonary rehabilitation program, in addition to general rehabilitation programs, would be beneficial for stroke patients.

This study has some potential limitations. First, the effects of the pulmonary rehabilitation program were monitored only at one and three months. A one-year follow-up would have been useful to shed light on the long-term effects of the PPT program on the prognosis. Second, the duration of PPT program was short and evaluation of pulmonary function tests before and after the PPT program were missing. A more effective PPT program considering its duration, intensity and frequency should be explored in the future. Third, the data were obtained from a single hospital setting and may not be generalizable to other settings. Finally, the effect of the infarct sizes, treatment regimens and information on patients’ function before hospitalization on the prognoses were not studied.

The strengths of the study were that it included an examination of multiple factors that may affect the prognosis of stroke patients. In addition, the patients’ disability scores were evaluated, and the same physician monitored all the patients.

## Conclusions

This study showed that a PPT program was effective in improving the prognosis of ischemic stroke patients. We believe that pulmonary rehabilitation programs, in addition to general stroke rehabilitation programs, can play a critical role in improving the survival and functional outcomes of acute stroke patients.

## Abbreviations

CI: Confidence interval
CT: Computed tomography
DWI-MRI: Diffusion-weighed magnetic resonance imaging
ICU: Intensive care unit
MCA: Medial cerebral artery
MRS: Modified Rankin scale
NIHSS: National Institute of Health Stroke Scale
OR: ODDS ratio
PPT: Pulmonary physiotherapy
TOAST: Trial of Org 10172 Acute Stroke Treatment
